# Effectiveness of emergency water treatment practices in refugee camps in South Sudan

**DOI:** 10.2471/BLT.14.147645

**Published:** 2015-06-01

**Authors:** Syed Imran Ali, Syed Saad Ali, Jean-Francois Fesselet

**Affiliations:** a University of California-Berkeley, 100 Blum Hall, MC 5570, Berkeley, CA 64720, United States of America (USA).; b University of Toronto, Toronto, Canada.; c Médecins Sans Frontières-Holland, Amsterdam, Netherlands.

## Abstract

**Objective:**

To investigate the concentration of residual chlorine in drinking water supplies in refugee camps, South Sudan, March–April 2013.

**Methods:**

For each of three refugee camps, we measured physical and chemical characteristics of water supplies at four points after distribution: (i) directly from tapstands; (ii) after collection; (iii) after transport to households; and (iv) after several hours of household storage. The following parameters were measured: free and total residual chlorine, temperature, turbidity, pH, electrical conductivity and oxidation reduction potential. We documented water handling practices with spot checks and respondent self-reports. We analysed factors affecting residual chlorine concentrations using mathematical and linear regression models.

**Findings:**

For initial free residual chlorine concentrations in the 0.5–1.5 mg/L range, a decay rate of ~5x10^-3^ L/mg/min was found across all camps. Regression models showed that the decay of residual chlorine was related to initial chlorine levels, electrical conductivity and air temperature. Covering water storage containers, but not other water handling practices, improved the residual chlorine levels.

**Conclusion:**

The concentrations of residual chlorine that we measured in water supplies in refugee camps in South Sudan were too low. We tentatively recommend that the free residual chlorine guideline be increased to 1.0 mg/L in all situations, irrespective of diarrhoeal disease outbreaks and the pH or turbidity of water supplies. According to our findings, this would ensure a free residual chlorine level of 0.2 mg/L for at least 10 hours after distribution. However, it is unknown whether our findings are generalizable to other camps and further studies are therefore required.

## Résumé

**Objectif:**

Enquêter pour déterminer la concentration en chlore résiduel des approvisionnements en eau potable de plusieurs camps de réfugiés, au Sud Soudan, entre mars et avril 2013.

**Méthodes:**

Pour chacun des trois camps de réfugiés étudiés, nous avons mesuré les caractéristiques physiques et chimiques des approvisionnements en eau, à quatre points de mesure à partir du point de distribution : (i) directement aux bornes-fontaines ; (ii) après la collecte ;(iii) après le transport jusqu'aux ménages ; et (iv) après plusieurs heures de stockage par les ménages. Les paramètres suivants ont été mesurés : chlore résiduel libre et chlore résiduel total, température, turbidité, pH, conductivité électrique et potentiel d'oxydoréduction. Nous avons documenté les pratiques de manipulation de l'eau à l'aide de contrôles par sondages et grâce aux auto-déclarations des personnes enquêtées. Nous avons analysé les facteurs affectant les concentrations en chlore résiduel à l'aide de modèles mathématiques et de régression linéaire.

**Résultats:**

Pour des concentrations initiales en chlore résiduel libre comprises entre 0,5 et 1,5 mg/L, une vitesse de dégradation d'environ 5x10-3 L/mg/min a été observée dans tous les camps. Les modèles de régression ont montré que la dégradation du chlore résiduel était liée aux concentrations initiales en chlore, à la conductivité électrique et à la température de l'air. Le fait de couvrir les conteneurs de stockage de l'eau (mais aucune autre pratique en termes de manipulation de l'eau) a entraîné une amélioration des concentrations en chlore résiduel.

**Conclusion:**

Les concentrations en chlore résiduel que nous avons mesurées dans les eaux approvisionnées dans les camps de réfugiés au Sud Soudan ont été trop faibles. En guise d'essai, nous recommandons d'élever la teneur en chlore résiduel préconisée à 1,0 mg/L dans toutes les situations, qu'il y ait une épidémie de maladies diarrhéiques ou pas et quels que soient le pH et la turbidité des eaux alimentées. D'après nos résultats, cela permettrait de garantir un taux de chlore résiduel libre de 0,2 mg/L pendant au moins 10 heures après la distribution. Néanmoins, nous ne savons pas si nos résultats sont généralisables à d'autres camps. D'autres études sont donc nécessaires.

## Resumen

**Objetivo:**

Investigar la concentración de cloro residual en los suministros de agua potable en los campos de refugiados de Sudán del Sur, entre marzo y abril de 2013.

**Métodos:**

Para cada uno de los tres campos de refugiados, se midieron las características físicas y químicas de los suministros de agua en cuatro puntos después de la distribución: (i) directamente de las tomas de agua; (ii) después de la recogida; (iii) tras transportarla a los hogares; y (iv) tras varias horas de almacenaje en los hogares. Se midieron los siguientes parámetros: el cloro residual libre y total, la temperatura, la turbiedad, el pH, la conductividad eléctrica y el potencial de reducción de oxidación. Se documentaron las prácticas de tratamiento del agua con verificaciones y autoinformes de los encuestados. Se analizaron los factores que afectan a las concentraciones de cloro residual mediante modelos de regresión matemáticos y lineales.

**Resultados:**

Para las concentraciones iniciales de cloro residual libre en el rango 0,5–1,5 mg/l, se encontró una tasa de depreciación de ~5x10^-3^ l/mg/min en todos los campos. Los modelos de regresión mostraron que la depreciación del cloro residual estaba relacionado con los niveles de cloro iniciales, la conductividad eléctrica y la temperatura ambiente. Los niveles de cloro residual mejoraron tras cubrir los contenedores de agua, pero no tras aplicar otras prácticas de tratamiento del agua.

**Conclusión:**

Las concentraciones de cloro residual que se midieron en los suministros de agua en los campos de refugiados de Sudán del Sur eran demasiado bajas. Se recomendó provisionalmente que la directriz de cloro residual libre se incrementara a 1,0 mg/l en todas las situaciones, independientemente de los brotes de la enfermedad diarreica y del pH o la turbidez de los suministros de agua. De acuerdo con nuestros resultados, esto debería garantizar un nivel de cloro residual libre de 0,2 mg/l hasta al menos 10 horas después de la distribución. Sin embargo, no sabemos si nuestros resultados son generalizables a otros campos y, por lo tanto, se requieren más estudios.

## ملخص

**الغرض إجراء:**

استقصاء لتحديد تركيز الكلور المتبقي في مياه الشرب المتاحة في مخيمات اللاجئين بجنوب السودان في الفترة من مارس إلى إبريل عام 2013.

**الطريقة:**

تولينا قياس الخواص الفيزيائية والكيميائية للمياه المتاحة في كل من ثلاثة مخيمات للاجئين خلال أربع مراحل بعد التوزيع: من الصنبور العمومي مباشرةً؛ 2) وبعد تجميع المياه؛ 3) وبعد نقل المياه إلى الأسُر؛ 4) وبعد عدة ساعات من التخزين لدى الأسُر. وتم قياس الخصائص التالية: نسبة الكلور الحر المتبقي وإجمالي الكلور المتبقي، ودرجة الحرارة، ونسبة التعكر، ودرجة الحموضة، والقدرة على توصيل الكهرباء، واحتمال الحد من الأكسدة. كما عملنا على توثيق ممارسات التعامل مع المياه من خلال بعض عمليات الفحص العشوائي والإبلاغ الذاتي المقدم من المستجيبين. وحللنا العوامل المؤثرة على تركيزات بقايا الكلور باستخدام أسلوب التحوّف الخطي والنماذج الرياضية.

**النتائج:**

بالنظر إلى ****التركيزات الأولية للكلور الحر المتبقي في نطاق 0.5 – 1.5 ملغ/لتر، تم اكتشاف معدل تحلل يبلغ ~5×10 ^-3 ^ لتر/ملغ/دقيقة وذلك في جميع المخيمات. وأظهرت نماذج التحوّف ارتباط تحلل الكلور المتبقي بمستويات الكلور الأولية، والقدرة على توصيل الكهرباء، ودرجة حرارة الهواء. وأدت تغطية حاويات تخزين المياه إلى تحسين مستويات الكلور المتبقي، على النقيض من الإجراءات الأخرى المتبعة للتعامل مع المياه.

**الاستنتاج:**

كانت تركيزات الكلور المتبقي التي قمنا بقياسها في المياه المتاحة في مخيمات اللاجئين بجنوب السودان شديدة الانخفاض. ونوصي مبدئيًا بزيادة النسبة المحددة للكلور الحر المترسب في المبدأ التوجيهي الخاص به إلى 1.0 ملغ/لتر في جميع الحالات، بغض النظر عن تفشي حالات الإصابة بمرض الإسهال ودرجة حموضة المياه المتاحة أو نسبة التعكر بها. وطبقًا للنتائج التي توصلنا لها، سيؤدي ذلك إلى ضمان وصول مستوى الكلور الحر المتبقي إلى 0.2 ملغ/لتر بعد توزيع المياه بعشر ساعات على الأقل. وعلى الرغم من ذلك، فلم نتمكن من معرفة ما إذا كانت النتائج التي توصلنا إليها يمكن تعميمها في المخيمات الأخرى، ومن ثم تدعو الحاجة إلى إجراء المزيد من الدراسات.

## 摘要

**目的:**

调查 2013 年 3 月至 4 月期间，南苏丹难民营饮用水供应的残留氯浓度。

**方法:**

在三个难民营中，我们逐个测量了配给后四个供水点的物理和化学特征： (i) 直接从水龙头收集； (ii) 收集之后； (iii) 输送至家家户户之后；以及 (iv) 在家中存储数小时之后。 我们测量了以下参数： 游离性残留氯和总残留氯、温度、浊度、pH 值、电导率和氧化还原电位。 我们采用抽查和受访者自我报告的形式对水处理实践进行记录。 我们使用数学和线性回归模型分析了影响残留氯浓度的因素。

**结果:**

初始游离性残留氯浓度在 0.5–1.5 毫克/升的范围内，我们发现在整个难民营内，衰减率为 ~5x10^-3^ 升/毫克/分钟。 回归模型显示，残留氯的衰减与初始氯含量、电导率和空气温度相关。 覆盖水存储容器，而非其它水处理实践提高了残留氯的含量。

**结论:**

通过对南苏丹难民营供水的测量，我们得出该地供水的残留氯浓度过低**。** 我们建议在不考虑腹泻病爆发以及 pH 值或者供水浊度的情况下，暂时将所有情形下游离性残留氯指标提高到 1.0 毫克/升。 根据我们的研究结果，这将确保水配给后至少 10 小时内游离型残留氯含量保持在 0.2 毫克/升。 但是，我们的研究结果是否适用于其他难民营尚不明确，因此还需要进一步的调查研究。

## Резюме

**Цель:**

Определение концентрации остаточного хлора в запасах питьевой воды в лагерях беженцев, Южный Судан, март-апрель 2013 г.

**Методы:**

Были проанализированы физические и химические свойства запасов воды в трех лагерях беженцев. Анализ воды проводился в четыре момента времени после ее доставки: (i) непосредственно у кранов с водой, (ii) после сбора, (iii) после транспортировки в дома и (iv) спустя несколько часов хранения в доме. Измерялись следующие характеристики: свободный и общий остаточный хлор, температура, мутность, уровень pH, электрическая проводимость и окислительно-восстановительный потенциал. Методы обработки воды документально фиксировались путем выборочных проверок на месте и личных опросов. Факторы, влияющие на концентрацию остаточного хлора, анализировались с использованием математических моделей и моделей линейной регрессии.

**Результаты:**

Для начальной концентрации свободного остаточного хлора в пределах 0,5–1,5 мг/л во всех лагерях отмечалась скорость разложения, примерно равная 5*10^-3^ л/мг/мин. Регрессионные модели показали, что разложение остаточного хлора было связано с начальным уровнем концентрации хлора, электрической проводимостью и температурой воздуха. Из всех методов хранения воды улучшить концентрацию остаточного хлора позволяло только закрывание контейнеров.

**Вывод:**

Концентрация остаточного хлора, измеренная в запасах воды в лагерях беженцев в Южном Судане, была слишком низкой. В порядке эксперимента предлагается увеличить рекомендуемое содержание свободного остаточного хлора до 1,0 мг/л во всех ситуациях без учета вспышек диарейных заболеваний, уровня pH или мутности потребляемой воды. Согласно результатам исследования, это позволит обеспечить концентрацию свободного остаточного хлора, равную 0,2 мг/л, на протяжении как минимум 10 часов после доставки воды. Тем не менее нельзя с уверенностью сказать, применимы ли результаты данного исследования к другим лагерям, поэтому необходимо проведение дополнительных исследований.

## Introduction

The late 19th and early 20th century saw rapid declines in mortality in industrialized countries. The introduction of chlorinated piped water supplies in cities was a major contributor to this achievement.^1^ Today, chlorination is the most widely-used method for the treatment of piped water supplies, due to its simplicity, low cost and the residual protection it provides.^2^^,^^3^ Low levels of residual chlorine in water supplies limit microbial contamination during distribution and storage, reducing the risk of waterborne infectious diseases. Drawing on decades of experience with municipal piped water systems around the globe, the World Health Organization (WHO) guidelines for drinking water quality recommend a minimum concentration of 0.2 mg/L free residual chlorine at water system delivery points.^4^

Humanitarian agencies generally use centralized batch chlorination for water treatment in settlements for refugees and internally displaced persons.^5^ This treatment method entails dosing an experimentally-determined amount of chlorine solution into a known volume of clear water, and allowing adequate retention time to allow disinfection to proceed to completion. Ensuring access to adequate quantities and quality of water is essential in refugee camps as waterborne diseases are among the most significant threats facing displaced populations.^6^^–^^9^ Drawing on WHO guidelines for drinking water quality, humanitarian organizations have developed several guidelines stipulating what residual chlorine levels should be at camp water distribution points.^10^^–^^16^ Generally speaking, guidelines recommend free residual chlorine levels should be 0.2–0.5 mg/L under normal circumstances and 0.5–1.0 mg/L during outbreaks of diarrhoeal disease, or when the water supply is especially turbid or alkaline. A balance is required between having sufficient residual protection and preventing taste and odour-driven rejection due to excessive chlorination.

WHO guidelines for drinking water quality are appropriate when users drink directly from the flowing household taps of a municipal piped water system,^17^ but are unlikely to provide sufficient residual chlorine protection in the fundamentally different reality of a refugee camp. In this setting, where environmental hygiene may be poor, water is collected from tapstands, transported in containers through the camp to shelters and then stored and used over 24 hours or more. Chlorine treatment based on WHO guidelines for drinking water quality may not ensure that water remains safe over its entire course in the setting of a refugee camp.

Studies in non-emergency settings in developing countries have shown recontamination of previously safe water does occur during collection and transport from distribution points, as well as during storage and drawing of water in the home,^18^^–^^21^ representing a significant health risk to vulnerable populations.^22^^,^^23^ Recontamination after collection of drinking water has also been documented in refugee camps in Uganda^24^ and linked to the spread of diarrhoeal disease and cholera among camp populations in Malawi,^25^^,^^26^ Kenya^27^^,^^28^ and Sudan.^29^ Humanitarian guidelines call for facilities and practices to preserve the safe water chain including the use of covered narrow-mouthed water containers with taps and their regular cleaning, disinfection and replacement. However, recontamination after distribution in camp settings remains poorly understood and is not explicitly included in guidelines for water treatment in emergency settings.

Recent experiences in refugee camps in South Sudan brought this knowledge gap to our attention. Surveys conducted in the Jamam camp in October–November 2012 showed that 40–58% of households that collected water from chlorinated tapstand sources had no detectable residual chlorine in their stored household water.^30^^,^^31^ Another study carried out in Jamam and the nearby Batil camp in April 2013 found adenoviruses in stored household water, suggesting faecal contamination.^32^ These observations, taken in light of the prolonged hepatitis E and acute watery diarrhoea outbreaks affecting the Maban County refugee camps,^33^ raised pressing questions about chlorination in camp settings.

Previous work has investigated and modelled how residual chlorine decays within water distribution systems.^34^ However, as far as we are aware, residual chlorine decay after water leaves the tap of the distribution system has not been investigated. We sought to: (i) investigate residual chlorine decay after distribution in the refugee camp setting and (ii) identify factors that preserve or compromise the safe water chain by exploring how water quality, water handling practices and contextual factors influence residual chlorine decay. In this paper, we investigate the effectiveness of emergency water treatment practices in the field. We contribute to the evidence base on water, sanitation and hygiene in emergencies and make recommendations for best practice.^35^^,^^36^

## Methods

### Study setting

The study was carried out at Jamam, Batil and Gendrassa refugee camps in Maban County, South Sudan during March–April 2013. At the time of the study, the population at these camps was 15 500, 37 200 and 15 800, respectively. The local climate and terrain in Maban County exacerbated the crisis and impeded response. The region is part of the Nile basin floodplain and characterized by thick strata of clay-rich soil. This soil is prone to water-logging, heavy and difficult to work with and unproductive with respect to groundwater. The rainy season runs from May to October and the dry season from November to April. The rainy season of 2012 saw the camps flooding, with latrines overflowing and inundating the surface, leading to multiple waterborne infectious disease outbreaks.^33^ Limited water availability, poor sanitation coverage and poor environmental hygiene exacerbated the outbreaks (Table 1).

**Table 1:** Water supply and sanitation in the three refugee camps, South Sudan, June 2012 to March 2013

Indicator by refugee camp	Sphere target	2012								2013		
		Jun	Jul	Aug	Sep	Oct	Nov	Dec		Jan	Feb	Mar
**Jamam**												
Population	–	31 686	30 277	21 179	16 751	13 984	15 439	15 765		15 765	15 765	15 670
Water supply, L/person/day	≥ 20	*7.5*	*7.5*	*11.3*	*17*	23.3	22.8	*18.8*		*13.8*	*17.5*	*18.9*
Water access, users per tap	≤ 80	*293*	*276*	*151*	*111.5*	*89*	NA	NA		*101*	*101*	*97*
Sanitation access, users per latrine	≤ 20	*37*	*39*	*23*	*22*	17	18	16		15.8	16	20
**Batil**												
Population	–	NA	NA	NA	NA	NA	NA	37 199		37 199	37 199	37 199
Water supply, L/person/day	≥ 20	NA	NA	NA	NA	NA	NA	*17.7*		*16.5*	*19*	*19.3*
Water access, users per tap	≤ 80	NA	NA	NA	NA	NA	NA	*85*		*86*	*86*	*84*
Sanitation access, users per latrine	≤ 20	NA	NA	NA	NA	NA	NA	20		*24*	18	19
**Gendrassa**												
Population	–	0	0	6 248	12 904	14 443	14 638	14 711		14 946	14 946	15 810
Water supply, L/person/day	≥ 20	NA	NA	*9.9*	*11.6*	*11.5*	*15.3*	*19.6*		21.2	21.0	25.6
Water access, users per tap	≤ 80	NA	NA	*86*	*130*	*104*	*92*	*90*		*87*	*87*	*88*
Sanitation access, users per latrine	≤ 20	NA	NA	18	*30*	16	12	17		15	15	14

NA: not available.

Note: Figures in italics indicate coverage below Sphere targets for each indicator. Indicators without available data are due to gaps in monitoring or because the camp was not yet established. Per person water supply figures assume 15% system loss.

Source: Médecins Sans Frontières.^37^

Groundwater was pumped from boreholes in or near the camps, treated with in-line chlorination then stored in tanks before being piped to tapstands for distribution. Tapstands in each camp were provided within 500 m of shelters, in accordance with Sphere Project guidelines.^16^ Supply was intermittent with water delivered for several hours in the morning and afternoon. Retention times in storage tanks following chlorination varied, contributing to variation in residual chlorine at tapstands. Water systems in all camps were in a state of flux at the time of the study, with elements being added or removed as populations fluctuated. Finally, given the outbreaks affecting the camps, outbreak protocols had been adopted and agencies aimed to deliver, in this case, 0.8–1.0 mg/L free residual chlorine at distribution points.

### Study design

We sought to follow the pathway of water in the camp setting from distribution at the tapstand to consumption at the household level. The study had two elements: (i) water quality analyses; and (ii) surveys of water handling practices and contextual factors. We assessed water quality at four points after distribution:

- analysis event 1: directly from the tapstand (event 1);
- analysis event 2: from users’ containers immediately after collection at the tapstand (event 2);
- analysis event 3: from containers after transport to shelters (event 3); and
- analysis event 4: from containers after several hours of household storage and use (event 4).

We analysed the following water quality parameters: free and total residual chlorine (the parameters of primary interest); pH and turbidity (both known to reduce chlorine disinfection efficiency);^2^^,^^38^ oxidation reduction potential (a proxy for disinfection potential);^39^ electrical conductivity (reflects the dissolved solids or salt content of drinking water); and air and water temperature (which affect the rate of chemical reactions).^40^ Quality control was achieved by calibrating analytical equipment every 1–2 days using manufacturer calibration standards.

We documented water collection, transport and storage practices, as well as other contextual factors, via spot checks and respondent self-reports at the tapstand (i.e. event 2) and in the household at follow-up (i.e. event 4). As it was crucial to follow the same unit of water, we also noted if the water had been transferred between containers, if it had been used (and, if so, how much) and whether it had been mixed with any other water at any time. The detailed study design is available from the corresponding author.

We initially set out to sample water from every borehole in each camp, however we found that boreholes were not always operational or residual chlorine was not always present at tapstands. Accordingly, we adopted a convenience sampling approach so that we could maximize data collection in the limited time available at each camp. We sought to capture spatial representativeness by: (i) visiting tapstands dispersed across the camp areas; and (ii) sampling water from tapstands attached to different boreholes. We approached whoever was collecting water at the tapstand for enrolment in the study. In total, we collected 220 unique samples roughly divided between the three camps. The study was submitted for ethics review to the Medical Director of Médecins Sans Frontières Operational Centre Amsterdam who exempted it from full ethics review as only routine operational data was being collected.

### Data analysis

We adopted a pooled data approach and modelled free residual chlorine concentration in MATLAB 7.12 (MathWorks Inc., Natick, United States of America) using the general integrated rate law:(1)
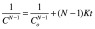
where *C_o_* is the initial chlorine concentration*, C* is the chlorine concentration at time *t*, *N* is the order of the reaction, and *K* is the rate constant.

We stratified by camp and by initial free residual chlorine concentration due to the nonlinearity of chlorine decay.^41^^–^^43^ Having estimated the model parameters *K* and *N*, we calculated the initial free residual chlorine levels at the tapstand that would ensure the desired level of free residual chlorine after storage for a defined period.

### Regression models

To explore associations between residual chlorine decay and water related physical and chemical parameters, handling practices and contextual factors, we used regression models in Stata 12.1 (StataCorp, College Station, USA). Regression models were run separately to investigate variables affecting water quality during three distinct stages: (i) during water collection; (ii) during transport to the household and (iii) during household storage and use (Table 2). Further details are available from the corresponding author.

**Table 2:** Variables included in regression models for water quality, South Sudan, March–April 2013

Stage	Variables included in regression model
During water collection from tap to container	Water quality at tap: free residual chlorine, turbidity, pH, water temperature, electrical conductivity
	Camp identity
	Tap type on the tapstand
	Observation of hand contact with water during collection
	Container type
	Container covering
	Container cleanliness
	Ambient air temperature
During transport from tapstand to the household	Water quality at tap: free residual chlorine, turbidity, pH, water temperature, electrical conductivity
	Camp identity
	Container type
	Container covering
	Container cleanliness
	Ambient air temperature
	Distance from tapstand to household
During household storage and use	Water quality at tap: free residual chlorine, turbidity, pH, water temperature, electrical conductivity
	Camp identity
	Container type
	Container covering
	Container cleanliness
	Ambient air temperature
	Method of drawing water
	Elapsed storage time
	Water transferred between containers
	Original water mixed with other water
	Water was used in the household

## Results

**Fig. 1:**
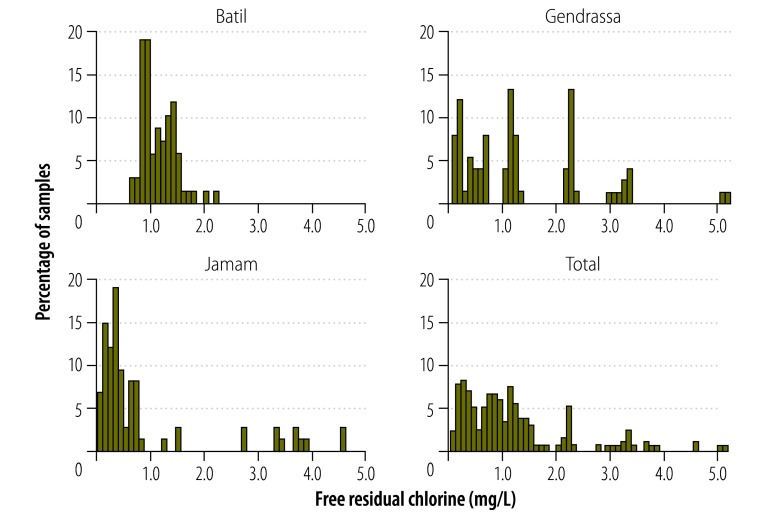
Histogram of free residual chlorine concentrations at the tapstand in refugee camps, South Sudan, March–April 2013

Note: Free residual chlorine concentrations ranged from just detectable (i.e. 0.01 mg/L) to excessive (i.e. 5.2 mg/L), reflecting poor chlorination performance of camp water systems.

Summary statistics on water quality at the tapstands, in each of the three camps, are given in Table 3. Residual chlorine levels were similar in all camps. The turbidity was below the upper limit for effective chlorination at all sites (5 nephelometric turbidity units, NTU). Water temperatures averaged greater than 30 °C at tapstands in the morning – an indication of how hot March and April are in this setting. The pH of the water at all camps was below 8.0, the upper limit for effective chlorination. Oxidation reduction potential varied widely between camps, partially reflecting residual chlorine levels but also potentially undocumented factors. Electrical conductivity, a proxy for chemical quality, was statistically different across camps (*P* < 0.0001) suggesting that each camp’s source was unique. As discussed earlier, chlorination performance at each site was less than ideal. A histogram of free residual chlorine concentrations encountered at tapstands is presented in Fig. 1.

**Table 3:** Water quality measurements at tapstands for the three refugee camps, South Sudan, March–April 2013

Parameter by refugee camp	*n*	Mean (SD)	Range
**Jamam**			
Free residual chlorine, mg/L	75	0.9 (1.2)	0.01–4.60
Turbidity, NTU	75	3.4 (2.0)	0.2–8.8
Water temperature, °C	74	32.0 (1.0)	29.2–34.1
pH	75	7.3 (0.8)	2.3–7.8
Oxidation reduction potential, mV	75	500 (158)	197–821
Electrical conductivity, mS/cm	75	1.7 (0.8)	0.2–2.0
**Batil**			
Free residual chlorine, mg/L	69	1.2 (0.3)	0.6–2.3
Turbidity, NTU	69	1.4 (1.3)	0.01–8.77
Water temperature, °C	69	31.1 (1.8)	27.3–37.6
pH	69	7.2 (0.5)	4.4–7.7
Oxidation reduction potential, mV	69	701(78)	342–861
Electrical conductivity, mS/cm	58	0.9 (0.3)	0.1–1.5
**Gendrassa**			
Free residual chlorine, mg/L	76	1.4 (1.2)	0.1–5.2
Turbidity, NTU	76	1.4 (0.9)	0.01–3.88
Water temperature, °C	76	30.2 (0.9)	27.8–32.4
pH	75	6.8 (0.7)	3.4–8.9
Oxidation reduction potential, mV	76	604 (124)	379–845
Electrical conductivity, mS/cm	60	0.6 (0.2)	0.4–1.0

NTU: Nephelometric turbidity units; SD: standard deviation.

Note: As sampling was initiated when free residual chlorine was detectable at tapstands, zero values were not captured in the data set and therefore the sample cannot be taken as representative of chlorination performance in the camps.

### Modelling residual chlorine

**Fig. 2:**
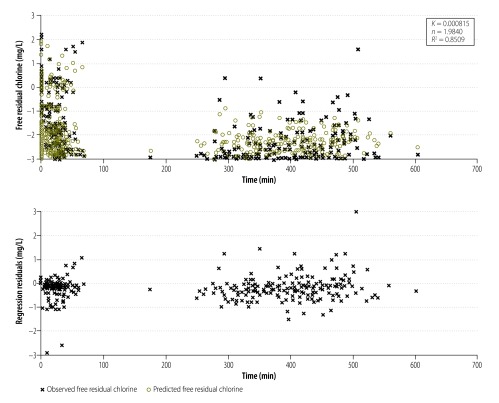
Decay model for free residual chlorine in refugee camps, South Sudan, March–April 2013

As seen in Table 4, modelling suggested that residual chlorine decay was a second-order process.^41^^–^^43^ The average model *R^2^* was 0.76 (range: 0.57–0.95). Examining modelling graphs and residual plots offers additional insight regarding goodness of fit. The pattern of the residuals was consistent across models, so the most general model (including data for all camps and all initial free residual chlorine data) is shown in Fig. 2.

**Table 4:** Modelling of initial free residual chlorine in refugee camps, South Sudan, March–April 2013

Concentration by refugee camp	No. of samples				*K* (L/mg/min)	*N*	*R^2^*
	Event 1	Event 2	Event 3	Event 4			
**All camps**							
All samples	220	186	199	205	8.150 × 10^−4^	1.98	0.85
0.2–1.0 mg/L	106	90	97	97	6.390 × 10^−3^	2.08	0.80
0.2–2.0 mg/L	166	142	151	153	3.824 × 10^−3^	2.03	0.74
**Jamam**							
All samples	75	68	69	71	6.630 × 10^−4^	2.00	0.95
0.2–1.0 mg/L	47	45	44	44	1.393 × 10^−2^	2.05	0.82
0.2–2.0 mg/L	50	48	47	47	7.048 × 10^−3^	2.07	0.77
**Batil**							
All samples	69	52	58	66	2.777 × 10^−3^	1.96	0.59
0.2–1.0 mg/L	30	20	25	28	3.656 × 10^−3^	2.15	0.57
0.2–2.0 mg/L	67	51	57	64	3.174 × 10^−3^	1.97	0.60
**Gendrassa**							
All samples	76	66	72	68	6.440 × 10^−4^	1.97	0.81
0.2–1.0 mg/L	29	25	28	25	6.537 × 10^−3^	2.06	0.87
0.2–2.0 mg/L	49	43	47	42	5.061 × 10^−3^	2.02	0.71

Note: Modelling was stratified by camp and initial free residual chlorine concentration. *K* is the rate constant and *N* is the order of the reaction.

We calculated the initial free residual chlorine concentrations required to ensure a desired level of free residual chlorine at a designated time after distribution (Table 5). A primary target of 0.2 mg/L at 24 hours after distribution was selected,^44^ with a secondary target of 0.2 mg/L at 12 hours. Table 5 reports projections using modelling outputs from the 0.5–1.5 mg/L initial free residual chlorine strata, as this is the range most relevant to field practice.

**Table 5:** Projections of free residual chlorine using decay models, South Sudan, March–April 2013

*C_o_* (mg/L)	Camp	*K* (L/mg/min)	*N*	*R^2^*	*C_o_* (mg/L) for a free residual chlorine of 0.2 mg/L after 12 hours	Time taken (h) for free residual chlorine to fall to 0.2 mg/L given *C_o_* = 1 mg/L
0.5–1.5	All	5.149 × 10^−3^	1.97	0.60	0.84	12.6
	Jamam	3.997 × 10^−3^	2.16	0.74	0.37	19.7
	Batil	4.998 × 10^−3^	1.94	0.59	0.86	12.5
	Gendrassa	5.884 × 10^−3^	1.92	0.51	2.19	10.4

Note: *K* is the rate constant; *N* is the order of the reaction; *C_o_* is the initial free residual chlorine concentration.

According to our model results, the primary target of 0.2 mg/L at 24 hours after distribution could not be achieved. For the secondary target, 0.2 mg/L at 12 hours after distribution, the initial concentration of free residual chlorine averaged 1.1 mg/L (range 0.37–2.19). If free residual chlorine at the tapstand was set to 1.0 mg/L, the residual concentration remained above 0.2 mg/L for an average of 13.8 hours (range 10.4–19.7; Table 5).

Linear regression models were used to explore relationships between free residual chlorine decay and water quality, water handling practices and contextual factors. These variables accounted for only about 25% of the variance in the data, suggesting that other unknown or undocumented factors are also important. Ambient air temperature during water collection was positively associated with free residual chlorine decay, suggesting that where ambient temperatures are high we can expect decay to be accelerated.^40^ There was some evidence of a direct relationship between decay and electrical conductivity, a proxy measure for dissolved metals and salts content of water, raising the possibility that complete oxidation of dissolved metals and other compounds, as required for effective chlorination and maintenance of residual, was not being achieved before distribution. Covering household water storage containers had a protective effect, while there was inconsistent evidence for other hygienic water handling practices (e.g. container cleanliness and method of drawing water). Detailed regression results are available from the corresponding authors.

## Discussion

According to our models, under the conditions in the South Sudan camps, it is not possible to ensure 0.2 mg/L of free residual chlorine 24 hours after distribution. Therefore, we must accept a lower level of protection than is ideal, or consider improvements to current practice. Centralized batch chlorination may be less appropriate where populations are dispersed across rural areas or within existing urban settlements; among populations with low chlorine taste or odour acceptance; or during the transitional phase from an acute to a stabilized emergency.^45^ In some situations, chlorine decay is so rapid that alternatives or adjuncts such as point-of-use water treatment may need to be considered.^46^

Regarding the safe water chain, covering household water storage containers was confirmed to be protective while there was weak or inconsistent evidence on the effect of other important hygienic water handling practices. The fact that an effect was not observed in the present study does not imply that these practices are necessarily ineffective; promotion of hygienic water handling practices remains an essential component of emergency safe water supply. Although there was some evidence concerning the effect of container and tap type, the study was not sufficiently powered to draw strong conclusions in this regard.

Current guidelines for free residual chlorine in emergency water supplies are not based on field evidence and offer inadequate protection after distribution in refugee camps in South Sudan. We recommend that the free residual chlorine guideline be increased to 1.0 mg/L in all situations, irrespective of disease outbreak, pH, or turbidity conditions. This is a tentative recommendation because the degree to which these findings can be generalized to other camps in different settings is unknown. According to our findings, an initial concentration of 1.0 mg/L will provide 0.2 mg/L free residual chlorine protection for at least 10 hours after distribution. This is consistent with the recommended concentration for point-of-use water chlorination in emergency and non-emergency settings^44^^,^^47^ and is within the limits generally considered to be acceptable to users (2.0 mg/L).^44^ Further studies are required in diverse climatic and environmental settings to expand the evidence base.
